# On the Treatment of Paraphymosis

**Published:** 1842-08

**Authors:** J. Togood

**Affiliations:** Bridgewater


*On the treatment of Paraphymosis.* By J. Togood, Esq., of Bridgewater.—The incision of the stricture in paraphymosis, is pretty generally practised by surgeons, except when this disease occurs in children, and in such the reduction is often effected by pressure. I have seen repeated incisions fail of relieving the stricture, and leave foul and intractable sores, which have been extremely tedious and difficult to heal. I have always succeeded in reducing a paraphymosis, either in an adult or child, however long standing, without having recourse to the knife, by the following method:—I place the patient against a wall, and take care that he is steadily supported by an assistant on each side; a piece of linen cloth is then laid over the glans, and with my thumbs I knead the blood out of it, drawing the prepuce forwards at the same time with two fore fingers of each hand. A steady perseverance in this plan never fails, and, although the operation is a painful one, the patient is amply rewarded by the rapidity of the cure, which requires nothing more than the application of a saturnine lotion for two or three days.—*Prov. Med. and Surg. Jan.* 15, 1842.

